# Meningitis-Retention Syndrome as a Presentation of West Nile Virus Meningitis

**DOI:** 10.1155/2013/984345

**Published:** 2013-07-31

**Authors:** Charoen Mankongpaisarnrung, Pavis Laengvejkal, Erwin Argueta, Chok Limsuwat, Grerk Sutamtewagul, Kenneth Nugent

**Affiliations:** Department of Internal Medicine, Texas Tech University Health Sciences Center, 3601 4th Street, Lubbock, Texas 79430, USA

## Abstract

A 26-year-old previously healthy man presented with fever, urinary retention, nuchal rigidity, and hyperreflexia but with a clear sensorium. His initial spinal fluid results were consistent with aseptic meningitis from West Nile virus infection, and this was confirmed by serological studies on blood and cerebrospinal fluid. Computed tomography and magnetic resonance imaging studies were unremarkable. He received supportive care and urinary catheterization to prevent bladder injury from overdistension. He was discharged home without recurrence of urinary retention after five days of hospitalization. Therefore, this case report describes the first case of West Nile virus meningitis in a patient with the meningitis-retention syndrome.

## 1. Introduction

West Nile virus is one of the mosquito-borne arboviruses in the *Flaviviridae* family. The clinical manifestations range from no symptoms in most cases to encephalitis in patients who have West Nile virus neuroinvasive disease. The first case was reported in a febrile woman in 1937 in Uganda. Since then, it has spread to the Middle East and Europe and now into the United States. In 2012, there was a large outbreak in the United States, specifically in Mississippi, South Dakota, Michigan, California, Louisiana, Oklahoma, Illinois, and Texas. We are reporting the first case of West Nile virus meningitis with acute urinary retention, consistent with the meningitis-retention syndrome. 

## 2. Case Presentation

**Figure 1:**
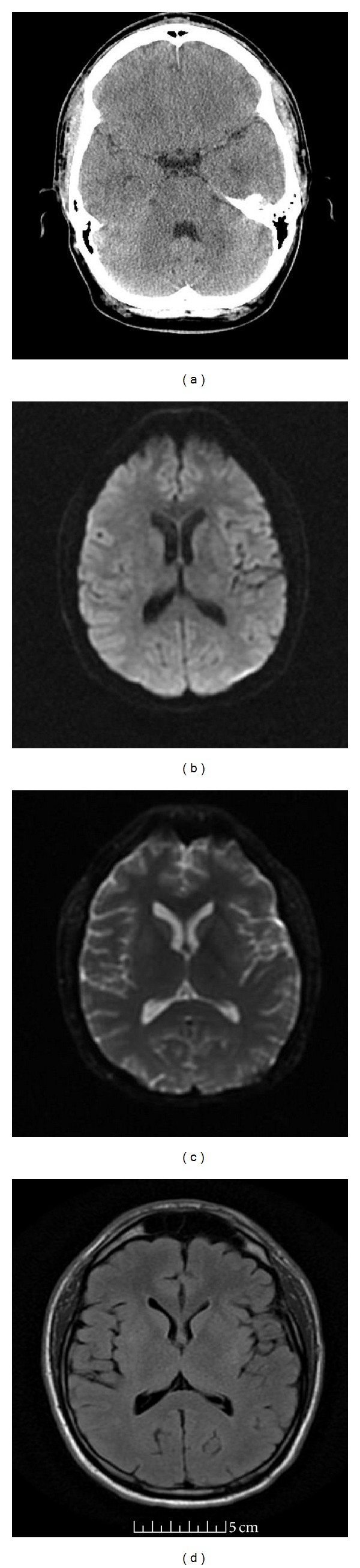
CT scan of brain without contrast was unremarkable (a) and axial MRI of the brain (DWI, ADC map, and FLAIR, resp., (b), (c), and (d)) at the level of basal ganglion was unremarkable.

**Figure 2:**
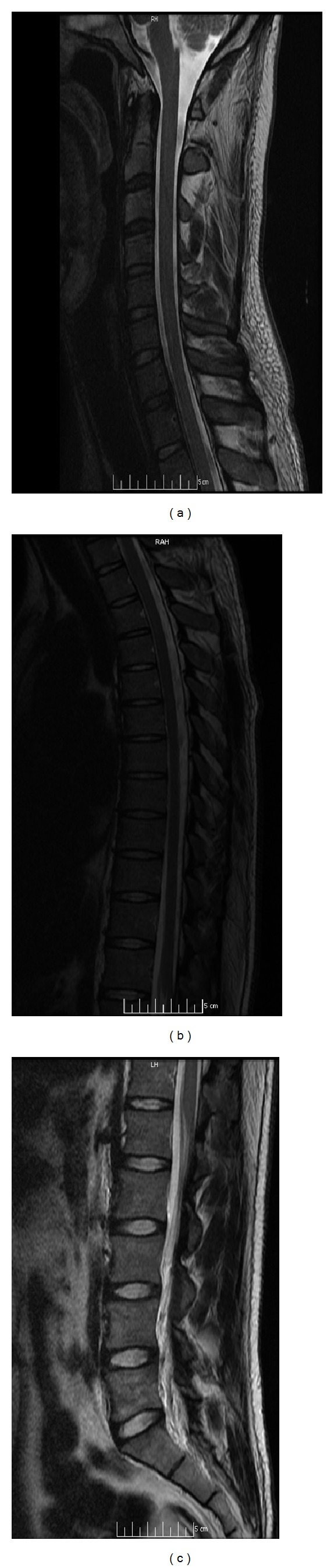
Sagittal MRI of the cervical, thoracic, and lumbosacral spinal cord (T2W; (a) to (c)) was unremarkable without a demonstrable compressive lesion.

A previously healthy 26-year-old man presented with high-grade fever, chills, headache with photophobia, nausea and vomiting, severe body aches, and a rash for a week after being in Denton, Texas, USAA. He then started to have suprapubic pain and acute urinary retention for two days. He was recently diagnosed with *Streptococcal *pharyngitis, and he developed a rash after amoxicillin was given. His treatment was then switched to azithromycin for three days. Serology for EBV infection was negative. Initially, his vital signs were temperature 103.9°F, BP129/85 mmHg, RR 22/min, and HR 80/min. He was acutely ill and was shivering. A maculopapular rash was present over his body and both cheeks. A full urinary bladder was also appreciated. A detailed neurological examination was normal except for nuchal rigidity and hyperreflexia. He had normal rectal sphincter tone and no sensory level. Transurethral bladder catheterization drained 1400 mL of urine. Computed tomography of the brain was unremarkable (Figure 1(a)). Lumbar puncture was performed with opening pressure of 16 cm H_2_O and closing pressure of 12 cm H_2_O. CSF analysis showed elevated WBC 225/mm^3^ (neutrophil 54%, lymphocyte 45%, and monocyte 1%), RBC 20/mm^3^, elevated protein of 115 mg/dL (15–45), and a normal glucose of 60 mg/dL (45–80). The ESR was 23 mm/hr, but the CRP was 56 mg/L. He was admitted to MICU with the diagnosis of aseptic meningitis. Broad-spectrum antibiotics and antivirals (meropenem, vancomycin, and acyclovir) were started promptly. Magnetic resonance imaging (MRI) of the brain and spinal cord was performed for acute urinary retention workup but was unremarkable (Figures 1(b)–1(d) and Figures 2(a)–2(c)). His HIV antibody was negative, syphilis IgG was negative and serum cryptococcal antigen was negative. West Nile virus serology results were IgG 1.07 (negative < 1.30) and IgM 4.64 (positive > 1.10). Cerebrospinal fluid for West Nile virus IgM was 6.83 (positive > 1.10), and IgG was 1.03 (negative < 1.30). Therefore, West Nile virus meningitis was diagnosed. The antibiotics were discontinued. The bladder training was begun; his urinary retention resolved and Foley catheter was discontinued upon day three of hospitalization. He was discharged home after five days of hospitalization without a catheter and no urinary retention. The patient was seen in clinic one week after discharge. He had a generalized mild headache, back pain, and weakness, but his overall condition had improved.

## 3. Discussion 

West Nile virus is one of the arboviruses and is transmitted to humans through the* Culex* mosquito vector. The reservoir for the West Nile virus includes many species of birds; humans and horses are only accidental hosts. The incubation period is between 2 and 15 days, and the clinical presentation ranges from no symptoms to debilitating symptoms with residual neurological deficits. Most people (70–80%) infected with West Nile virus are asymptomatic. Only 20–30% of infected patients will develop West Nile fever, and less than 1% of infected patients will develop the West Nile Virus neuroinvasive disease. This can cause encephalitis, aseptic meningitis, and poliomyelitis-like syndrome with acute asymmetrical flaccid paralysis in the limbs. Elderly patients are more likely to develop West Nile virus encephalitis. Distinctive features of West Nile virus neuroinvasive disease include muscle weakness in 30–50% of those with encephalitis and lower motor neuron involvement, such as facial paralysis. Rashes develop in 20–30% of patients [1–3]. 

Unusual presentations of West Nile virus infection include respiratory failure secondary to diaphragmatic paralysis, fulminant hepatic failure, pancreatitis, myocarditis, arrhythmia, myositis, orchitis, nephritis, and optic neuritis. Urinary retention can also occur and has been reported as a urologic complication in the setting of encephalitis [4]. However, there has never been a case reported with isolated acute urinary retention with meningitis. The primary treatment of WNV infection is supportive care; treatable causes of aseptic meningitis should be treated empirically during workup or until confirmation of the diagnosis.

We are reporting the first case of West Nile virus meningitis presenting with acute urinary retention consistent with meningitis-retention syndrome. His clinical presentation and radiographic studies are compatible with the meningitis-retention syndrome. The diagnosis of West Nile virus meningitis was confirmed by serological and cerebrospinal fluid studies. This syndrome is an underrecognized clinical constellation of aseptic meningitis and acute urinary retention, and it carries a favorable prognosis. Most patients with meningitis-retention syndrome recover from urinary retention within three weeks, and it is currently considered a self-remitting disease without sequelae. The initial three case reports in the literature were described by Sakakibara in 2005 [5]. This report included two men and one woman with an age range of 34–68. They had mononuclear pleocytosis in the CSF with an elevated protein and normal to near normal glucose levels. All viral studies were negative. MRI studies of the brain and spinal cord were normal, and the urinary retention resolved within three weeks.

Myelin basic protein was found in the cerebrospinal fluid in one of the initial cases, and this suggests that these patients may have demyelination in the central nervous system. This led to the hypothesis that meningitis-retention syndrome represented a “very mild” form of acute disseminated encephalomyelopathy (ADEM) or sacral myeloradiculitis from a postinfectious inflammatory demyelinating response in the peripheral and central nervous system [6]. ADEM is an immune-mediated inflammatory and demyelinating disorder of central nervous system characterized by a widespread demyelination that predominantly involves the white matter of the brain and spinal cord [7], which is commonly preceded by an infection. It typically occurs within 2 days to 4 weeks after a viral infection or less commonly after vaccination [8]. Patients with meningitis-retention syndrome have an adynamic detrusor muscle, which could reflect injury to the sacral cord (level S2 and S3) or the parasympathetic pelvic ganglia. Some patients have hyperdynamic internal urinary sphincters that would require involvement of either the spinal cord (T11-S2) or lower abdominal sympathetic ganglia. Most meningitis-retention syndrome patients have an unremarkable MRI of the head and spinal cord, but there is a report of abnormal MRIs with reversible posterior splenial lesions seen in this patient group [9]. In addition, Sakakibara reported that MRI studies revealed spotty T2 high intensities in the basal ganglion, thalamus, and brainstem and enhancement of conus medullaris and cauda equina with gadolinium [10]. 

Hyperreflexia was a prominent physical finding in our case and suggests mild pyramidal tract involvement. This physical finding and the unremarkable MRI finding made sacral myeloradiculitis unlikely. Consequently, patients with meningitis-retention syndrome likely have abnormal parasympathetic innervation of the detrusor muscle. The bladder is initially areflexic but can become hyperreflexic during recovery [11]. The sequence is similar to spinal shock.

 The Elsberg syndrome was first described in 1931 as subacute to chronic radiculitis with acute urinary retention secondary to lumbosacral myeloradiculitis of unclear etiology. Possible causes include vasculitis, ADEM, viral infection, such as Herpes simplex virus type 2, 6, and *Angiostrongylus cantonensis *infection [9]. There are also case reports suggesting that bacterial infections with *Listeria monocytogenes* and *Neisseria gonorrhoeae* can be associated with acute urinary retention in conjunction with acute meningitis [12]. The Elsberg syndrome was initially regarded as myeloradiculitis resulting in acute urinary retention with possible lumbosacral muscular weakness from cauda equina syndrome in association with local viral infection/reactivation, such as genital Herpes infection, without the clinical picture of meningitis. The terminology of “meningitis-retention syndrome” has been recently used to describe the combined features of symptomatic aseptic meningitis with acute urinary retention without apparent local viral infection/reactivation [13]. In some cases, Herpes simplex virus has been considered the cause of Elsberg syndrome since this virus can remain in a latent form in the nerve root ganglion, but Herpes virus was not recovered in CSF studies in reported patients [14]. These associations suggest that Elsberg syndrome and meningitis-urinary retention syndrome represent either direct microbial infection of the central or peripheral nervous system or an immune-inflammatory response secondary to infection with subsequent injury to the spinal cord or peripheral nerves.

In summary, meningitis-retention syndrome is an uncommon syndrome with aseptic meningitis and acute urinary retention. These patients do not have encephalitis or myelitis; this presentation may represent a mild form of ADEM. The management of this syndrome requires prevention of bladder injury from overdistension with the use of indwelling catheter. The role of corticosteroid treatment remains unproven as an effective measure. In our patient, his urinary retention resolved soon after his hospitalization and did not recur.
